# Amylose *amyloid light-chain* (*AL*) et lymphome folliculaire: à propos d’un cas

**DOI:** 10.11604/pamj.2024.49.128.43592

**Published:** 2024-12-18

**Authors:** Amine Benmoussa, Hajar Maatoui-Belabbes, Reda Allali, Meriem Regragui, Meriem Qachouh, Siham Cherkaoui, Mouna Lamchahab, Mohamed Rachid, Abdellah Madani, Nisrine Khoubila

**Affiliations:** 1 Service d’Hématologie Clinique, Faculté de Médecine et de Pharmacie Hassan II, Centre Hospitalier Universitaire Ibn Rochd Casablanca, Casablanca, Maroc,; 2 Service d´Anatomie Pathologique, Faculté de Médecine et de Pharmacie Hassan II, Centre Hospitalier Universitaire Ibn Rochd Casablanca, Casablanca, Maroc

**Keywords:** Amylose *AL*, lymphome folliculaire, évolution, traitement, cas clinique, AL amyloidosis, follicular lymphoma, progression, treatment, case report

## Abstract

L´association de l´amylose AL et le lymphome folliculaire B est extrêmement rare car le clone secrétant la chaine légère amyloïdogène est généralement plasmocytaire. Nous rapportons le cas d´un patient âgé de 67 ans sans ATCDs pathologiques particuliers ayant présenté une altération de l´état général avec une dysphonie et dysphagie d´aggravation progressive avec une volumineuse masse du cavum dont la biopsie était en faveur d´un lymphome non hodgkinien folliculaire B grade 1 et 2. La tomodensitométrie (TDM) cervico-thoraco-abdomino-pelvienne a objectivé une masse nasopharyngée de 70 mm x 40 mm étendue sur 60 mm. La biopsie ostéomédullaire était normale ainsi que le bilan préthérapeutique. Le malade a reçu 4 cures de protocole Rituximab plus CHOP (cyclophosphamide, adriamycine, prednisone et oncovin) sans réponse puis 3 cures Rituximab plus DHAOX (dexamethasone, aracytine haute dose, et oxalipatine) avec persistance de la masse. La biopsie de cette dernière a objectivé la disparition de l´infiltration lymphoïde B avec présence des dépôts amyloïdes AL. L´immunoélectrophorèse des protéines plasmatiques a mis en évidence la présence de l´immunoglobuline M. La tomographie par émission de positons (TEP) scan a objectivé un processus nasopharyngé hypermétabolique. Le malade est actuellement sous protocole bortezomib, prednisone et bendamustine.

## Abstract (en)

The association of light-chain (AL) amyloidosis with type B follicular lymphoma is extremely rare, as the clone secreting the amyloidogenic light chain is generally plasmacytic. We here report the case of a 67-year-old patient with no specific pathological history, presenting with a deterioration in general condition, progressively worsening dysphonia and dysphagia, and a large mass in the cavum. Biopsy findings indicated grade 1-2 type-B follicular non-Hodgkin’s lymphoma. Cervico-thoraco-abdomino-pelvic computed tomography (CT) scan revealed a nasopharyngeal mass measuring 70 mm x 40 mm and extending over 60 mm. Bone marrow biopsy and pre-therapeutic evaluations were normal. The patient received 4 cycles of rituximab plus CHOP (cyclophosphamide, adriamycine, prednisone and oncovin) with no response, followed by 3 cycles of rituximab plus DHAOX (dexamethasone, high-dose cytarabine and oxalipatin) with persistence of the mass. A subsequent biopsy of the mass revealed the disappearance of B-cell lymphoid infiltration but showed AL kappa chain amyloid deposits. Immunoelectrophoresis of plasma proteins detected the presence of IgM kappa immunoglobulin. Positron emission tomography (PET) imaging identified a hypermetabolic nasopharyngeal process. The patient is currently undergoing treatment with a protocol combining bortezomib, prednisone, and bendamustine.

## Introduction

L´amylose *AL* est le plus souvent primitive parfois associée à un myélome multiple. Son association à certains types histopathologiques d´un lymphome non hodgkinien (LNH) B (lymphome lymphoplasmocytaire, le lymphome à cellules B de la zone marginale ganglionnaire/le lymphome MALT, la leucémie lymphoïde chronique/lymphome lymphocytique et d´autres) est rapportée dans la littérature [1-3]. Cependant, à notre connaissance, 2 cas d'amylose *AL* en association avec le lymphome folliculaire B ont été précédemment rapportés [4,5]. Nous présentons les caractéristiques cliniques et l´évolution d´un patient atteint d´une amylose *AL* associée à un lymphome folliculaire B suivi dans notre service d´hématologie clinique.

## Patient et observation

**Informations relatives au patient:** il s´agissait d´un patient âgé de 67 ans, sans antécédents pathologiques qui a consulté pour une gêne à la déglutition avec une dysphonie et une altération de l´état général.

**Figure 1:**
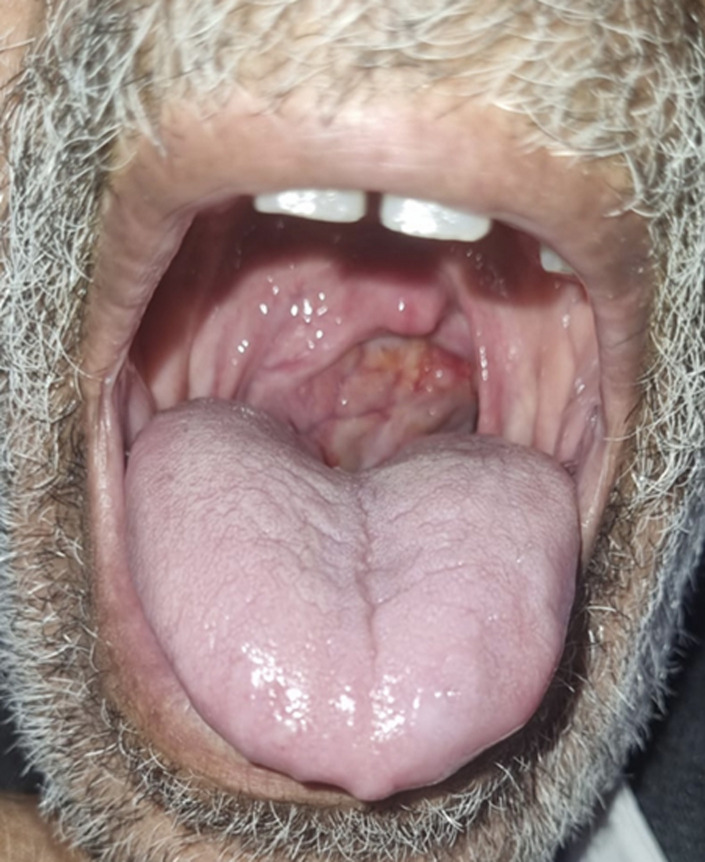
volumineuse masse au niveau du cavum

**Résultats cliniques:** l'examen clinique initial trouvait un patient conscient avec un score de Glasgow à 15/15, apyrétique, une tension artérielle de 12/07 cmHg, une saturation en oxygène de 100%, une fréquence cardiaque à 80/min, des conjonctives normocolorées avec la présence d´une volumineuse masse au niveau du cavum (Figure 1). Il n´y avait pas d´hépatomégalie ni de splénomégalie, les aires ganglionnaires étaient libres, le reste de l'examen somatique était normal.

**Chronologie:** le patient présentait depuis 6 mois une gêne à la déglutition avec une dysphonie, le tableau clinique s´est aggravé par l´installation d´une dysphagie aux solides avec une altération de l´état général (amaigrissement chiffré à 15kg/6 mois).

**Démarche diagnostique:** la TDM cervico-thoraco-abdomino-pelvienne a objectivé une masse nasopharyngée de 70 mm x 40 mm étendue sur 60 mm. Le bilan biologique du patient était normal (la numération de formule sanguine, le bilan rénal et hépatique, la lacticodéshydrogénase et les sérologies VIH VHC VHB). L´étude histologique et immunohistochimique de la biopsie nasopharyngée était en faveur de LNH folliculaire B grade 1,2 CD20+; CD19+; CD79a+; CD10+ sur 2 lectures dans deux laboratoires différents. La biopsie ostéomédullaire était normale ainsi que le bilan préthérapeutique.

**Intervention thérapeutique:** le patient a reçu 4 cures RCHOP 21 (rituximab 375mg/m^2^ en intraveineux (iv), cyclophosphamide 750 mg/m^2^ iv, oncovin 2 mg iv, prednisolone 100 mg par voie orale, et doxorubicine 50 mg/m^2^ (iv) sans réponse puis 3 cures RDHAOX (rituximab 375 mg/m^2^ en intraveineux (iv) à J1, aracytine haute dose 2 g/m^2^ x 2 iv à J2, dexamethasone 40 mg de J1 à J4, et oxalipatine 100 mg/m^2^ à J1) sans réponse clinique.

**Figure 2:**
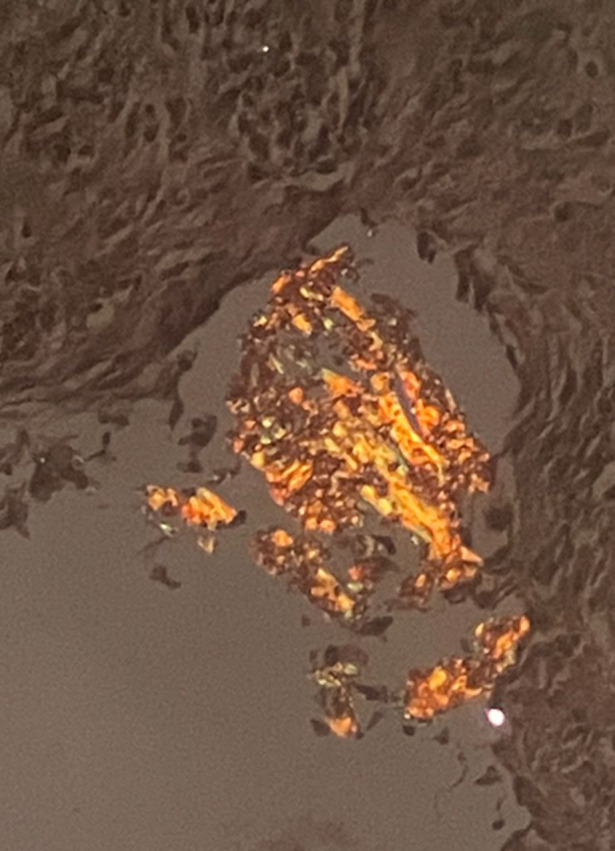
présence des dépôts éosinophiles amorphes exprimant une biréfringence vert pomme à la coloration rouge Congo en lumière polarisée

**Suivi et résultats des interventions thérapeutiques:** la persistance et l´augmentation de la masse nasopharyngée a conduit à la réalisation de la trachéotomie, la biopsie de la masse nasopharyngée a objectivé la disparition de l´infiltration lymphoïde B avec présence des dépôts amyloïdes *AL* type kappa (Figure 2).

L´immunoélectrophorèse des protéines plasmatiques a mis en évidence la présence de l´immunoglobuline M kappa, le dosage des chaines légères n´a pas été réalisé par manque de moyens, le myélogramme et une deuxième biopsie ostéomédullaire étaient normaux, la TEP scan a objectivé un processus nasopharyngé hypermétabolique sans autres anomalies, l´évaluation cardiaque (ECG, peptides natriurétiques, troponine, echocoeur) et rénale étaient sans particularités, le malade est actuellement sous protocole bortezomib, prednisone et bendamustine avec bonne évolution clinique après première cure.

**Point de vue du patient:** il a rapporté une nette amélioration après la première cure du protocole bortezomib, prednisone et bendamustine avec diminution de la taille de la masse nasopharyngée et la disparition de la dysphagie.

**Consentement éclairé:** le patient a donné son consentement pour publication de son cas.

## Discussion

L'amylose à chaînes légères (*AL*) est une maladie proliférative des plasmocytes monoclonaux rarement des lymphoplasmocytes ou lymphocytes caractérisés par la précipitation dans les tissus intercellulaires de chaînes légères monoclonales d´immunoglobuline sous forme de fibrilles qui résistent à la protéolyse, provoquant des perturbations mécaniques et un stress oxydatif local dans les organes affectés tels que le cœur, les reins, le foie et le tractus gastro-intestinal. Elle est le plus souvent associée à un néoplasme plasmocytaire sous-jacent, mais elle est rarement causée par un lymphome non hodgkinien (LNH) à cellules B. Les cellules lymphoïdes tumorales B en tant que l´origine productrice des chaînes légères d'Immunoglobulin amyloïdogènes n'ont été identifiées que dans environ 2% des cas d'amylose *AL* [6]. Lorsque l´amylose *AL* est associée à un LNH, une paraprotéine IgM est souvent présente. L´amylose IgM est le plus souvent associée au lymphome lymphoplasmocytaire/macroglobulinémie de Waldenström (74% des cas). Une association avec d'autres types de LNH est extrêmement rare et n'a été rapportée que dans de petites séries de cas [7].

La présentation des amyloses IgM se distingue de celle des non IgM par la fréquence moindre des atteintes cardiaques (32-45% dans l'amylose IgM contre 70% dans l'amylose non IgM), plus de localisations ganglionnaires ou pulmonaires, et l´implication plus fréquente d´une chaine légère kappa. Le seul fait d´avoir une IgM monoclonale associée à une amylose ne suffit pas pour conclure que le clone sécrétant l´IgM est lymphoplasmocytaire ou lymphocytaire. Il est fondamental de préciser au mieux la nature de la prolifération B monoclonale, par une étude précise de tissu pathologique, la moelle osseuse (et/ou des lymphocytes circulants) comportant un immunophénotypage et, si possible, une étude génétique [6,7]. Ce qui rend notre cas rare, c´est que nous avons découvert que seulement 2 cas de coexistence d´amylose *AL* et le lymphome folliculaire ont été rapportés dans la littérature, 1 seul cas dans une large série de cas française et un cas japonais avec une amylose *AL* et lymphome folliculaire avec différenciation plasmocytaire [4,5]. Notre patient est atteint d´amylose *AL* de type IgM localisée stade I associée au LNH folliculaire de forte masse tumorale.

Les patients atteints d'une amylose IgM à un stade précoce selon le score de la Mayo Clinic (stade I ou II) ont une survie plus faible que les patients atteints d'une forme non IgM à un stade précoce (75% de survie globale à 5 ans pour l´amylose IgM de stade 1 contre >90% pour la forme non IgM) et ont des taux de réponse au traitement plus faibles [8]. Le développement de traitements efficaces pour ce sous-type rare d'une maladie déjà rare est donc un défi, les décisions thérapeutiques reposent principalement sur des données rétrospectives. Notre patient avait une évolution défavorable avec persistance et augmentation en taille de la masse nasopharyngée malgré la disparition de l´infiltration lymphoïde tumorale initiale au niveau du cavum après chimiothérapie.

L'objectif du traitement est d'éliminer la production de la protéine amyloïde en ciblant le LNH à cellules B sous-jacent. Les traitements typiques des amyloses associées aux proliférations plasmocytaires ne sont pas efficaces dans ce sous type, car ils ne ciblent pas le clone tumoral lymphoïde [9].

L´amylose IgM a toujours été traitée par une chimiothérapie conventionnelle seule (par exemple, melphalan, cyclophosphamide), les régimes à base de rituximab associé au bortezomib, aux immunomodulateurs et aux inhibiteurs de Bruton tyrosine kinase (BTKi) ont été introduits dans des études rétrospectives et des séries de cas de petite taille [10]. Le traitement du premier cas de LNH folliculaire avec amylose *AL* publié dans la série française n'a pas été précisé, tandis que le deuxième cas japonais a subi une résection iléale puis il a reçu 6 cures de chimiothérapie R-THP-COP (rituximab, cyclophosphamide, pirarubicin, vincristine, et prednisone) avec rémission complète.

A l´heure actuelle, il n'existe aucune ligne directrice standard en matière de traitement, d´où l´intérêt de réaliser d´autres études de cas pour déterminer la meilleure stratégie thérapeutique possible.

## Conclusion

L´amylose *AL* est le plus souvent primitive ou associée à un myélome multiple, cependant son association à un lymphome non hodgkinien doit être recherchée. L´association de l´amylose *AL* et le lymphome folliculaire est rarement décrite dans la littérature, d'où l´intérêt d´autres études de cas pour clarifier la physiopathologie, les caractéristiques cliniques et évolutives et discuter les modalités thérapeutiques de cette association.
